# A Case of Chloroform Poisoning Treated by Hypodermic Injections of Whisky and Strychine—Recovery

**Published:** 1881-02

**Authors:** Robert W. Westmoreland

**Affiliations:** Atlanta, Ga.


### Medical and Surgical Journal.

VOL. XVIII.] FEBRUARY—1881. [No. 11.

### Original Communications.

### A CASE OF CHLOROFORM POISONING TREATED BY HYPODERMIC INJECTIONS OF WHISKY AND STRYCHNINE—RECOVERY.

BY ROBERT W. WESTMORELAND, M.D., ATLANTA, GA.

A double action is claimed for chloroform by some, based upon the fact that different modes of exhibition produce distinct physiological results. The action, for instance, obtained by inhalation, and that by internal administration, it is claimed, are as separate and distinct as if resulting from totally different agents. From inhalation we have produced first, a stage of temporary excitement, succeeded by one of depression. "In this the sensorium and the reflex organs are involved, until a state of complete unconsciousness and insensibility may finally be produced"—*Binz.* In this condition of uncomplicated anesthesia, Claude Bernard has directly proved a true anemia of the brain to exist.

On the other hand, given by the stomach or by the rectum, it has been conceded as conclusively demonstrated by Pirogroff, Marc Dupuy and others, as impossible to produce true and complete anesthesia. The physiological action attributed to it, however, by this mode of administration, is of the character rather between—so to speak, a cerebral stimulant and tonic. Of course there is a consecutive stage of more or less depression following this stimulant effect, but this is more analogous to the effect produced by the class of remedies to which opium belongs.

We cannot be actually certain, probably, of the difference in the pathological condition in a state of unconsciousness produced by an anesthetic or by such remedies as belladonna, opium, calabar bean, etc. The physical manifestations antecedent to the stage of complete insensibility, produced by these agents respectively, are very different, yet more than a superficial investigation is necessary to discriminate after the period of unconsciousness has set in.

During last summer I was called to see a woman who had, for the purpose of committing suicide, it was alleged, swallowed a large amount of chloroform. The exact amount could not be ascertained. Soon after she had taken it, Dr. Owen, of this place, was called in. The circumstances not having been fully stated to him, and he thinking nothing very serious had occurred, simply gave her an emetic. Soon afterward I was called to see her at the Station House. I found her in an unconscious and almost lifeless condition. The breathing was stertorous, while the pulse was between fifty and sixty. The strong odor of chloroform in the breath, in connection with a detail of the preceding circumstances, satisfied me that I had to deal with a case of chloroform poisoning. On the principle of the great reflex excitability and increase to the heart's action, physically produced by *caffein*, I promptly decided on its exhibition in this case. I succeded in getting two full doses down her throat, with an interval of an hour between them.

Some acceleration of the heart's action was the result, and also some improvement in respiration. As the time for the next dose came around, I attempted to give the medicine, but could not succeed so well as before, strangulation and suppression of the breathing resulting from the effort. Artificial respiration was instituted and not having an hypodernice syringe, I left to procure one, thinking, however, that it would hardly be of any avail.

While away she improved considerably, and upon my return I found her general condition better. At this time Drs. Owen and Nicholson having become associated in the case, on their suggestion we resorted to the hypodermic administration of strychnine and whisky. At first we introduced about 2 drachms of whisky, and ten drops of a solution of gr 1 of strychnine to one ounce of water.

This was repeated at the proper interval, and it was soon discovered that the pulse increased in frequency ; the temperature, which had been much below normal, (I forget the exact figures of the observation,) gradually increased, and evidences of returning consciousness were manifested. Soon the woman awoke from her torpor, and, with an eye single to the fixed resolve that had at first actuated her, she cried, "give me some more chloroform." The recovery was complete ! The whisky and strychnine, I am satisfied, snatched her from a death that would have been, but for them, inevitable.

Were I to attempt to offer a theory of the *modus operandi* of the action of these two remedies in effecting a recovery in this case, I would be inclined to place them, in reference to the acting poison Chloroform, in an analogous relation to that existing between bromide potassium in strychnine poisoning, or that which is supposed to exist between woorari in tetanus. It was incidently through their capability of awakening reflex iritability and cerebral activity that the patient recovered, and it has induced me to the belief that they constitute the *sine qua non* in the treatment of a case of chloroform poisoning by internal administration.

